# Complex application of bacteriophages as a method of healthcare-associated infections control

**DOI:** 10.1186/2047-2994-4-S1-P46

**Published:** 2015-06-16

**Authors:** E Brusina, O Drozdova, L Zueva, V Akimkin, L Fedorova

**Affiliations:** 1 Research Institute for Complex Issues of Cardiovascular Diseases, Russian Federation; 2 State Budget Educational Institution of Higher Professional Education “Kemerovo State Medical University”, Kemerovo, Russian Federation; 3 State Budget Educational Institution of Higher Professional Education «I.I. Mechnikov’s North Western State Medical University», Saint Petersburg, Russian Federation; 4 State Budget Educational Institution of Higher Professional Education “I.M. Sechenov’s First Moscow State Medical University”, Moscow, Russian Federation; 5 Federal Budget Scientific Institution “Scientific Research Disinfectology Institute” of Federal Service for Surveillance on Consumer Rights Protection and Human Well-being, Moscow, Russian Federation

## Introduction

Prevention of healthcare-associated infections is one of the global problems in modern period of healthcare system development.

## Objectives

Prophylaxis of HAI in Russia.

## Methods

Epidemiological, statistical.

## Results

Over the past 50 years in Russia, considerable progress in theoretical evidence and practical application of bacteriophages to treat and prevent healthcare-associated infections has accumulated. The efficient use of bacteriophages in epidemic outbreaks of healthcare-associated infections has been documented in numerous representative studies of different epidemiological centers in Russia: Kemerovo, St. Petersburg, Moscow, Nizhny Novgorod, Ufa and others.

One of the important areas of application is the use of bacteriophages for the decontamination of inanimate objects and surfaces in healthcare settings. Disinfection by using bacteriophages is the most suitable in epidemiologically relevant specialized departments such as intensive care units, burn units, etc. Drugs containing phages adapted to circulating bacterial strains will be more efficient compare to their non-adapted counterparts.

## Conclusion

Numerous randomized controlled trials revealed different efficacy regarding the usage of bacteriophages in the environment. The greatest effect was obtained using Pseudomonas bacteriophage: complete elimination of the pathogen was achieved within 24 hours after a single application, and there were no new cases of healthcare-associated Pseudomonas infections. This is also the case for Salmonella bacteriophage effect (15-fold incidence reduction).

## Disclosure of interest

None declared.

